# IL-33 induction and signaling are controlled by glutaredoxin-1 in mouse macrophages

**DOI:** 10.1371/journal.pone.0210827

**Published:** 2019-01-25

**Authors:** Ellen O. Weinberg, Beatriz Ferran, Yuko Tsukahara, Michaela M. S. Hatch, Jingyan Han, Colin E. Murdoch, Reiko Matsui

**Affiliations:** 1 Department of Medicine, Section of Infectious Diseases, Boston University School of Medicine, Boston, Massachusetts, United States of America; 2 Department of Medicine, Vascular Biology Section, Whitaker Cardiovascular Institute, Boston University School of Medicine, Boston, Massachusetts, United States of America; National Institutes of Health, UNITED STATES

## Abstract

Interleukin (IL)-33 is an interleukin-1 like cytokine that enhances Th2 responses and mediates mucosal immunity and allergic inflammation but the mechanism regulating endogenous IL-33 production are still under investigation. In macrophages, lipopolysaccharide (LPS) administration resulted in marked induction of IL-33 mRNA that was blunted in macrophages from glutaredoxin-1 (Glrx) knockout mice and in RAW264.7 macrophages with Glrx knockdown by siRNA. Glutaredoxin-1 is a small cytosolic thioltransferase that controls a reversible protein thiol modification, S-glutationylation (protein-GSH adducts), thereby regulating redox signaling. In this study, we examined the mechanism of Glrx regulation of endogenous IL-33 induction in macrophages. Glrx knockdown resulted in impaired de-glutathionylation of TRAF6, which is required for TRAF6 activation, and inhibited downstream IKKβ and NF-κB activation. Inhibitors of NF-κB suppressed IL-33 induction and chromatin IP sequencing data analysis confirmed that IL-33 is an NF-κB-responsive gene. Since TRAF6-NF-κB activation is also essential for IL-33 signaling through its receptor, ST2L, we next tested the involvement of Glrx in exogenous IL-33 responses in RAW264.7 cells. Recombinant IL-33 (rIL-33) administration induced IL-33 mRNA expression in RAW264.7 macrophages, and this was inhibited by Glrx knockdown. Interestingly, rIL-33-induced IL-33 protein was identified as the 20 kDa cleaved form whereas LPS-induced IL-33 protein was identified as full-length IL-33, which may be less active than the cleaved form. In a clinically-relevant mouse model of asthma, intra-tracheal cockroach antigen treatment induced Glrx protein in wild type mouse lungs but Glrx induction was attenuated in IL-33 knockout mouse lungs, suggesting that IL-33 may regulate Glrx induction *in vivo* in response to allergen challenge. In summary, our data reveal a novel mechanism by which Glrx controls both LPS- and IL-33-mediated NF-κB activation leading to IL-33 production, and paracrine IL-33 can induce Glrx to further regulate inflammatory reactions.

## Introduction

IL-33 is a member of the IL-1 family that was identified as the ligand of the orphan receptor, ST2L (formal name, IL-1 receptor-like 1, IL1RL1), and transduces signals through transmembrane ST2L receptor via TNF receptor-associated factor 6 (TRAF6) [1]. IL-33 is known to enhance Th2 responses and mediate allergic reactions [1,2]. Endogenous IL-33 contributes to innate-type mucosal immunity and allergic airway inflammation [3–5].

IL-33 is constitutively stored in the nucleus of certain barrier cell types including epithelial cells and endothelial cells [6–8], and in the later, may function as a transcriptional regulator [6,9] although a recent report refutes this [10]. *Alternaria alternata* exposure in the airways of mice and *in vitro* induces rapid release of IL-33 by epithelial cells into the extracellular milieu. In this setting, ATP mediated activation of purinergic receptors and sustained increases in calcium are responsible for IL-33 release both *in vitro* and *in vivo* [11].

IL-33 is not constitutively expressed in monocytes and macrophages, rather, it is induced by the bacterial endotoxin, lipopolysaccharide (LPS) [1,12–14] and is released from injured or dying cells [11,13,15]. In macrophages, IL-33 plays a role in polarization to alternatively-activated M2 macrophages that may be involved in wound healing [16–18]. A recent study showed that Ly6C-positive F4/80-positive monocytes population was a major source of IL-33 and contributed to allergic inflammation in the lungs following house dust mite stimulation [19], indicating that inducible IL-33 in monocytes may be an important player in allergic lungs. IL-33-deficient mice show diminished systemic inflammatory responses to LPS [3] while paracrine IL-33 enhances LPS-induced inflammatory cytokines in macrophages [20], suggesting interaction and/or augmentation in signaling between IL-33 and the LPS-toll like receptor (TLR) 4 pathway.

LPS also induces glutaredoxin-1 (Glrx), a small cytosolic thioltransferase that specifically reverses glutathione (GSH) adducts (*S*-glutathionylation) from protein thiols [21]. Glrx-mediated GSH-protein adduct reversal (de-glutationylation) is emerging as an important mechanism for cellular redox signaling including regulation of inflammatory pathways in a number of disease models and settings [22,23]. Glrx deletion enhances GSH adduct formation on key signaling proteins including those of the NF-κB pathway resulting in NF-κB inactivation by inhibiting IKKβ and p50 DNA binding [24,25] and decreased pro-inflammatory cytokine release in alveolar macrophages [21]. On the other hand, increased Glrx levels activate NF-κB through reversal of GSH adducts [26,27]. In the present study we examined the mechanism of Glrx regulation of IL-33 RNA and protein induction in macrophages. We report the novel finding that Glrx regulates both LPS-induced and IL-33-induced IL-33 production in macrophages via regulation of TRAF6-NF-κB activation; however, independent of Glrx regulation, IL-33-induced IL-33 protein was a processed short form that is likely to be more bioactive compared to LPS-induced IL-33. Finally, we provide *in vivo* evidence that our findings of reciprocal regulation between IL-33 and Glrx may be involved in a mouse model of asthma.

## Materials and methods

### Reagents

Lipopolysaccharide (L6529) was obtained from Sigma-Aldrich. Recombinant mouse IL-33 was from R&D Systems. Antibodies were obtained from the following sources; anti-mouse IL-33 (goat polyclonal, AF3626) was from R&D Systems, anti-TRAF6 (rabbit polyclonal) and anti-IκBα (rabbit polyclonal) were from Santa Cruz Biotechnology, anti-mouse Glrx (rabbit polyclonal, BL3725) was from IMCO/Cayman or custom-made by Bethyl Laboratories, Inc., anti-thioredoxin-1 was a generous gift from Dr. J. Sadoshima (Rutgers New Jersey Medical School). Rabbit monoclonal antibody to Phospho-IKKα/β (Ser176/180) was from Cell Signaling. MG132 was obtained from Cayman, and JSH-23 was from EMD Chemicals, Inc. High Capacity RNA-to-cDNA kit, TaqMan Gene Expression Master Mix, TaqMan assays and StepOne Real-Time PCR Systems were from Applied Biosystems Thermo Fisher Scientific. German cockroach antigen was obtained from Stallergenes Greer.

### Mouse peritoneal macrophages

Glrx knockout (KO) mice were generated by Dr. Y-S. Ho (Wayne State University, MI) [28]. Homozygous Glrx KO and genetically-matched wild type (WT) control mice were transferred and bred in the Laboratory Animal Science Center (LASC) on the Boston University Medical Campus as approved by Institutional Animal Care and Use Committee at Boston University. Mice were euthanized by carbon dioxide inhalation according to a protocol approved by Boston University Animal Care and Use Committee. Peritoneal macrophages were collected from WT and KO mice (8–10 months old) by injection of cold PBS containing glucose into the peritoneal cavity immediately after euthanasia and collection of peritoneal fluid. Cells from 4–5 mice were pooled and centrifuged at 1000 rpm, re-suspended in DMEM containing 10% FBS, and plated into 24 wells. After one hour, cells were washed with warm PBS to remove non-adherent cells and adherent cells were cultured in DMEM +10% FBS with penicillin-streptomycin [29]. The following day the medium was changed, and cells were treated with LPS (100 ng/ml) for 6 hours followed by RNA extraction. Before LPS stimulation an aliquot of cells was fixed with 4% paraformaldehyde and stained with anti-mouse F4/80 antibody (BioLegend). F4/80 positive cells assessed by Image J of total cell number which was counted by Hoechst staining,

### RAW 264.7 macrophage culture and Glrx knockdown by siRNA

Mouse macrophage cell line, RAW 264.7, was obtained from ATCC (TIB-71) and cultured in DMEM with 10%FBS and 4.5 mg/L glucose as instructed. To knock down expression of Glrx, approximately 5 X 10^5^ cells were plated in 6-well plates. Adherent cells were transfected with siRNA for mouse Glrx-1 (Invitrogen): sense: GCAGAAAGACCCAAGAAAU, antisense: AUUUCUUGGGUCUUUCUGC (Glrx siRNA) or scrambled RNA (Con siRNA) at a final concentration of 125 nM using Lipofectamine 2000 (Invitrogen) in the medium containing 10% FBS without antibiotics. After 2 days in culture the medium was replaced with DMEM containing 1% FBS, followed by treatment with LPS (100 ng/ml) or IL-33 (10–25 ng/ml). For some experiments, confluent cells were treated with LPS in DMEM containing 1% FBS after incubation with inhibitors for 30 minutes.

### Biotinylated GSH Ester (BioGEE) and detection of GSH-protein adducts

To detect GSH-protein adduct formation, the method originally described [30] was applied with slight modifications. Briefly, BioGEE was made by mixing EZ-Link Sulfo-NHS-Biotin (Pierce) with GSH ethyl ester in 50 mM NaHCO_3_ at pH 8.5 for 2 h followed by addition of 250 mM NH_4_HCO_3_ (pH 8.5) to quench remaining biotinylation reagent. Purified BioGEE was a generous gift from Dr. M. Bachschmid (Boston University). Confluent RAW264.7 cells, after Glrx siRNA or Con siRNA treatment, were incubated with 100 μM BioGEE in DMEM with 1% FBS for 2 hours with or without LPS. Cells were washed in HBSS with 100 mM *N*-ethylmaleimide for 5 minutes, followed by collecting in lysis buffer (Cell Signaling Technology #9803) containing 100 mM *N*-ethylmaleimide, 1% SDS, 1 mM phenylmethylsulfonyl fluoride. Cell lysates were sonicated on ice, incubated at 50°C for 20 minutes with shaking to block free thiols and centrifuged at 1000 rpm for 5 min. The supernatant was passed through a PD-10 Sephadex-G25 column to remove excess BioGEE, and an equal amount of eluted protein was mixed with streptavidin-Sepharose beads overnight at 4°C. Beads were washed with lysis buffer 4 times and the final precipitate was incubated in Laemmli buffer with 5% β-mercaptoethanol and 5M urea, followed by separation by SDS-PAGE. TRAF6 was detected by immunoblotting with polyclonal anti-TRAF6 antibody.

### RT-qPCR

Total RNA was isolated from macrophages using Trizol (Invitrogen) followed by DNase digestion. RNA was reversed transcribed to cDNA using High Capacity RNA to cDNA kit (4387406, Thermo Fischer Scientific). Relative quantitative PCR (qPCR) was performed using gene-specific TaqMan primers (Invitrogen). TaqMan assays used were: Glrx Mm00728386_s1, Il10 Mm01288386_m1, Il33 Mm00505403_m1, ST2L (Il1rl1) Mm01233982_m1. β-actin (mouse 4352933) was used to normalize the expression. Expression levels were analyzed by comparative Ct (ΔΔCT) with StepOne^TM^ real-time PCR software (Applied Biosystems).

### Western Blotting and ELISA

Protein concentration was assessed in cell lysates by Bio-Rad DC assay (500–0112, Bio-Rad Laboratories). Equal amounts of protein in each sample in Laemmli buffer were run on 4–12% SDS-PAGE gels. Proteins were transferred onto supported PVDF membranes and blocked with 3% milk. Primary antibodies were incubated overnight at 4°C in 3% albumin in PBST. Horseradish peroxidase (HRP)-conjugated secondary antibodies were used. Blots were imaged for chemiluminescence on films or by Kwik Quant Imager. Mouse IL-33 ELISA was purchased from eBioscience.

### Cockroach antigen-stimulation in IL-33 knockout mouse lungs

IL-33 KO mice and wild type control mice were generated as described elsewhere and genotype was confirmed by PCR (Smith J et al, submitted). Briefly, cockroach antigen (CRA, Greer Laboratories) was administrated intratracheally to produce allergic lung inflammation [31]. Lungs were harvested 4 hours after the last intratracheal administration. Expression of Glrx was performed using Western blotting.

### Statistics

Data are expressed as mean ± SE. Statistical comparisons were performed by Student’s t-test or two-way ANOVA followed by Bonferroni post-test. Statistical significance was accepted when P was less than 0.05.

## Results

### LPS-induced IL-33 gene expression was markedly inhibited in peritoneal macrophages from Glrx KO mice

**Fig 1:**
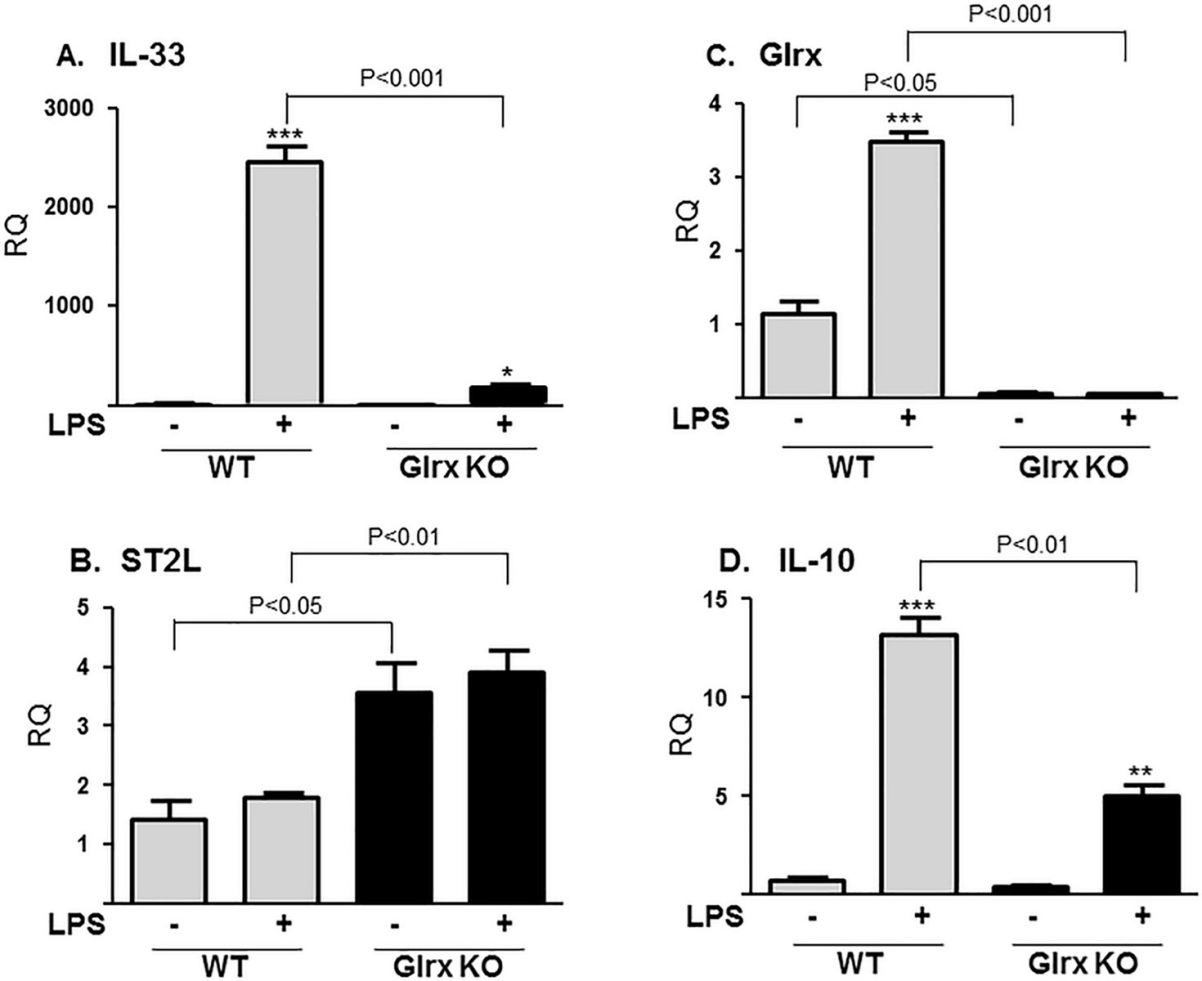
LPS-induced IL-33 gene expression was inhibited in peritoneal macrophages from Glrx KO mice. Isolated peritoneal macrophages from WT or Glrx KO mice were seeded overnight and stimulated with LPS (100 ng/ml) for 6 hours, followed by RNA isolation and RT-qPCR. Relative quantity (RQ) was standardized by β-actin expression. LPS-induced expression of (**A)** IL-33, (**B)** ST2L, (**C)** Glrx, and (**D)** IL-10, each were inhibited in Glrx KO macrophages compared to WT macrophages. Expression of ST2L, the receptor for IL-33, was higher in Glrx KO macrophages with or without LPS. Control (C) is no LPS. n = 3. ** *p*<0.01, *** *p*<0.001 show the difference between C vs LPS in the same genotype of mice (One-way ANOVA).

IL-33 gene expression following *in vitro* challenge with lipopolysaccharide (LPS) was examined in mouse peritoneal macrophages from Glrx KO and WT mice by relative quantitative RT-PCR and standardized by β-actin expression. Immunostaining of isolated peritoneal cells in culture showed 82% cells were positive for the macrophage marker, F4/80 (**S1 Fig**). After exposure to LPS (100 ng/ml) for 6 hours, IL-33 was highly induced by LPS in WT cells and this induction was strikingly inhibited in Glrx KO cells. (**Fig 1A)** WT basal expression equal to 1; WT + LPS was 2454 ± 168 and KO + LPS was 171 ± 26, n = 3, p<0.01). Expression of IL1RL1 (ST2L), which encodes the full-length transmembrane IL-33 signaling receptor, was not altered by LPS stimulation in WT cells but was significantly higher in Glrx KO cells (RQ: WT+LPS 1.79 ± 0.07, KO+LPS 3.92 ± 0.35, n = 3, p<0.05) (**Fig 1B**). Glrx was also strongly induced by LPS in WT cells and this induction was, as expected, absent in Glrx KO cells (**Fig 1C**). IL-10, which is known as an anti-inflammatory gene was also induced by LPS in WT cells and this induction was also inhibited in Glrx KO cells (**Fig 1D**). These data were further confirmed using RAW264.7 macrophages to examine Glrx-mediated regulation of signaling in macrophages.

### Glrx knockdown attenuated LPS-induced IL-33 in RAW264.7 mouse macrophages

**Fig 2:**
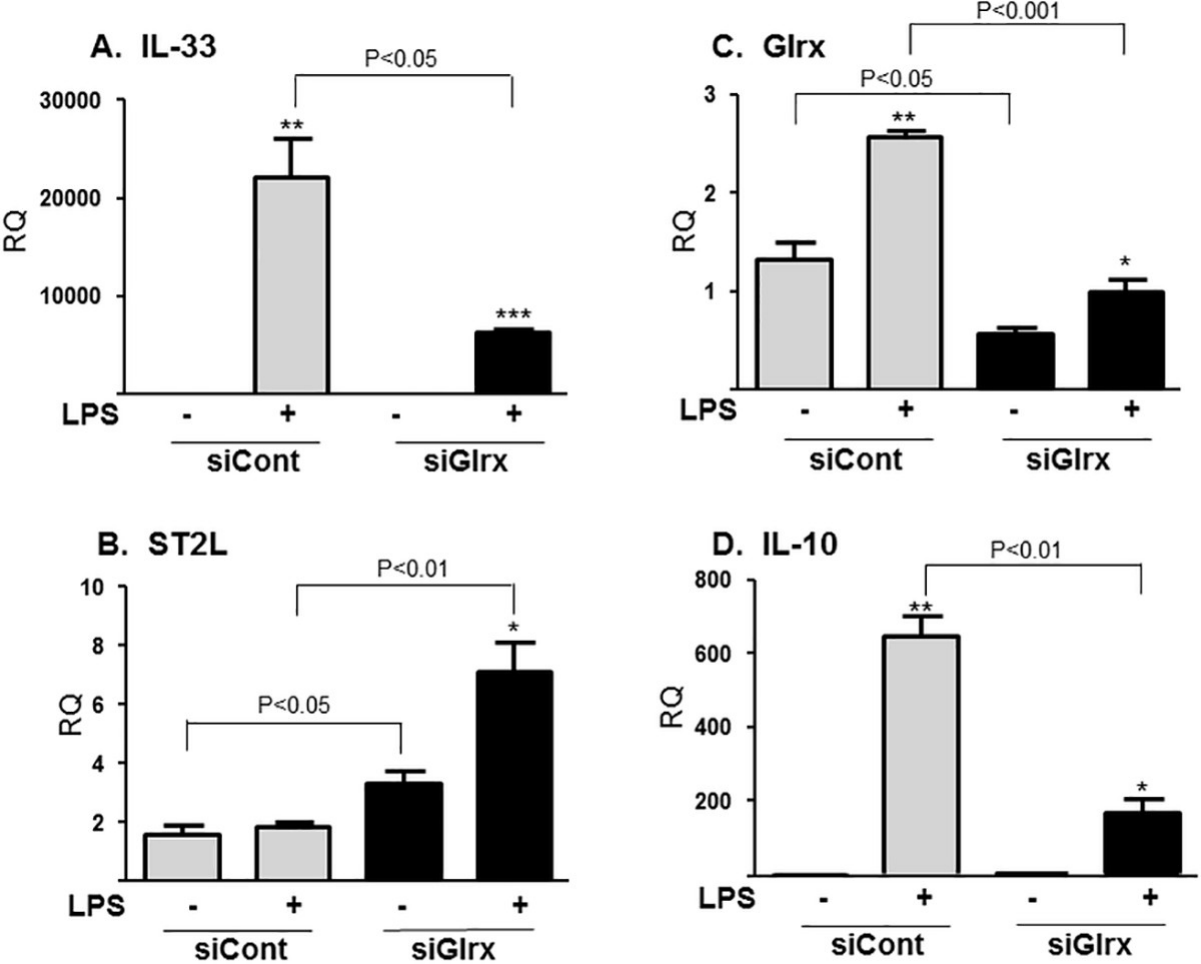
LPS-induced IL-33 expression in RAW267.4 macrophages with Glrx knockdown. RAW267.4 cells were treated with control siRNA (siCont) or Glrx siRNA (siGlrx). The media was replaced with 1% FBS media followed by stimulation with LPS (100 ng/ml) for 6 hours. Relative quantity (RQ) was assessed with β-actin and normalized by the basal level of siCont (n = 3, *p<0.05, ** *p*<0.01, *** *p*<0.001 show the difference between C vs LPS). The induction of (**A)** IL-33 was significantly inhibited, (**B)** ST2L expression was up-regulated by Glrx knockdown. (**C)** Glrx, (**D)** IL-10. Similar results were obtained in at least 3 independent experiments.

To examine signaling pathways in Glrx-regulated IL-33 induction, Glrx was knocked down by siRNA in RAW264.7 mouse macrophages. Comparable to our observations in peritoneal macrophages from Glrx KO and WT mice, LPS-induced IL-33 mRNA expression was significantly inhibited by Glrx knockdown (**Fig 2A**). In addition, ST2L mRNA expression was higher following Glrx knockdown compared to siCont in the absence of LPS, and its expression was further stimulated by LPS (**Fig 2B**); a finding that was different from the response in peritoneal macrophages. As was the case in peritoneal macrophages, IL-10 mRNA levels in response to LPS were also diminished following knockdown of Glrx (**Fig 2D**).

**Fig 3:**
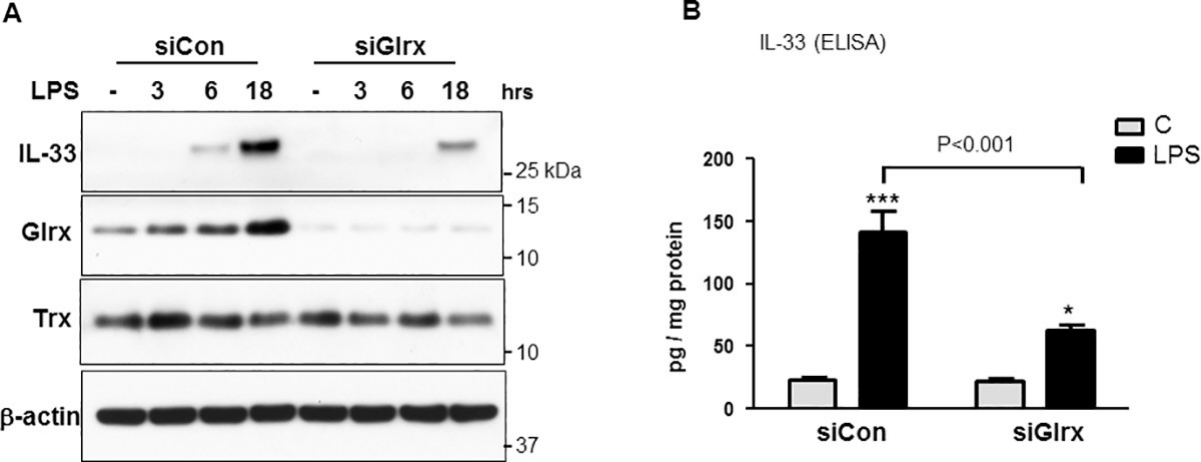
IL-33 protein levels were inhibited by Glrx knockdown. (**A)** IL-33 were assessed by Western blot in RAW267.4 cell lysates after stimulation with LPS (100 ng/ml) for 6–18 hours. β-actin expression is shown as a loading control. (**B)** IL-33 protein was also measured by ELISA after 18 hours LPS stimulation in RAW267.4 cells (n = 3, * *p*<0.05, *** *p*<0.001 from Control of each siRNA).

IL-33 protein levels were analyzed by Western blotting and ELISA using RAW264.7 cell lysates. LPS (100 ng/ml) treatment resulted in increased IL-33 as well as Glrx protein levels in a time-dependent manner up to 18 hours. Glrx knockdown strongly attenuated LPS-induced IL-33 protein expression detected by Western blotting (**Fig 3A**) as well as by ELISA (**Fig 3B**). Expression of thioredoxin, a thiol-reducing enzyme, was not changed by either LPS treatment or Glrx knockdown (**Fig 3A**).

### NF-κB is involved in IL-33 induction

**Fig 4:**
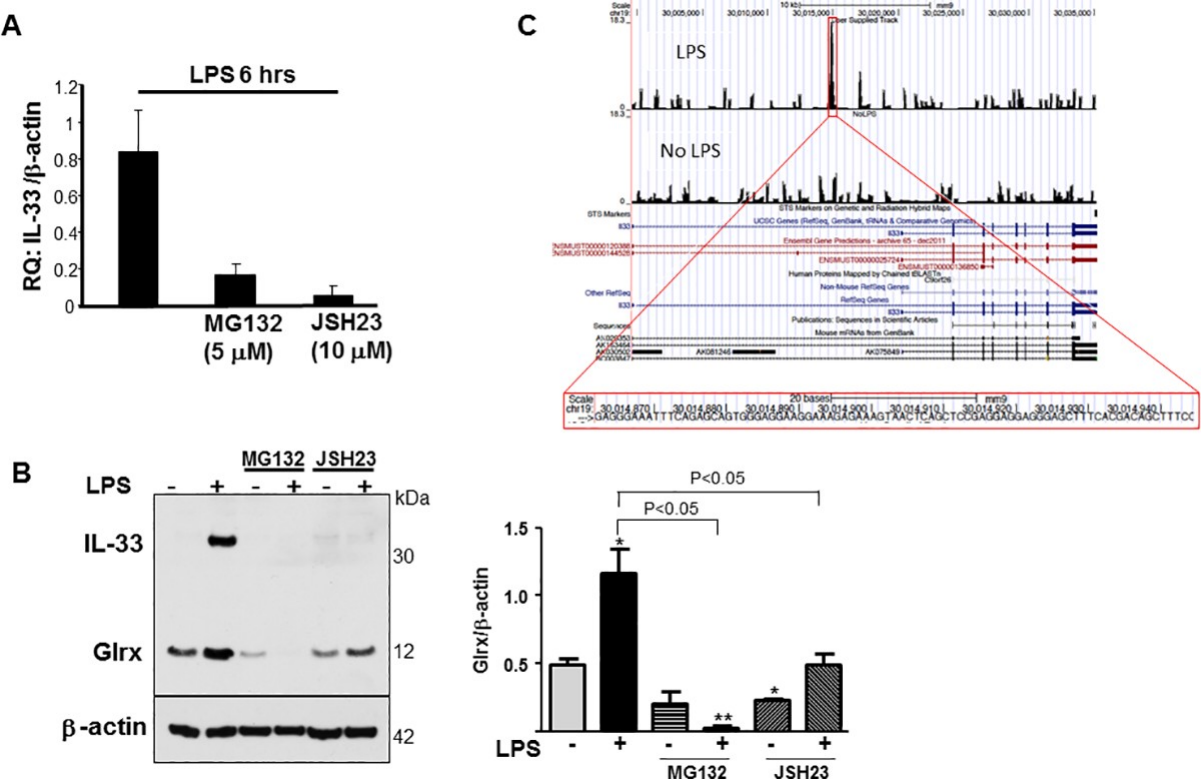
IL-33 gene is NF-κB responsive and has p65-binding site. Confluent RAW267.4 cells in 1%FBS media were treated with the NF-κB inhibitors, MG132 (10 μM) or JSH23 (5 μM, IC50 = 7.1 μM) for 30 minutes, followed by LPS (100 ng/ml) stimulation for 6 hours. (**A)** IL-33 mRNA levels. (**B)** Left: Representative Western blotting showing IL-33 protein induction was inhibited following treatment with NF-κB inhibitors. Glrx induction was also attenuated by the inhibitors. Right: Densitometry analysis of Glrx expression. (n = 3, *p<0.05, **p<0.01 compared to control) (**C)** ChIP seq data from macrophages treated with LPS followed by pulldown with p65 antibody was extracted from the data obtained by Barish GD et al. *Genes and Development* 2010 [32]. The IL33 gene nucleotide sequence residing on chromosome 19 was isolated and subjected to analysis using the UCSC Genome Browser. Highlighted in red (top) is the major peak in ChIP seq data resulting from treatment of macrophages with LPS that is not present in macrophages with no LPS treatment. Enlarged section of this region (bottom) shows approximately 80 nucleotide region that is the putative NF-κB (p65) responsive element.

To determine whether IL-33 is an NF-κB responsive gene in macrophages in response to LPS, we performed studies *in vitro* to analyze IL-33 induction using NF-κB inhibitors, MG132 and JSH23. These studies showed inhibition of LPS-induced IL-33 induction at mRNA (**Fig 4A**) and protein levels (**Fig 4B**). Also, ChIP seq data from macrophages treated with LPS followed by pulldown with p65 antibody was extracted from Barish GD et al [32] and subjected to analysis using the UCSC Genome Browser. A major peak on chromosome 19 and annotated as IL-33 was identified resulting from treatment of macrophages with LPS that was not present in macrophages without LPS treatment (**Fig 4C**).

### NF-κB pathway is inhibited by Glrx knockdown

**Fig 5:**
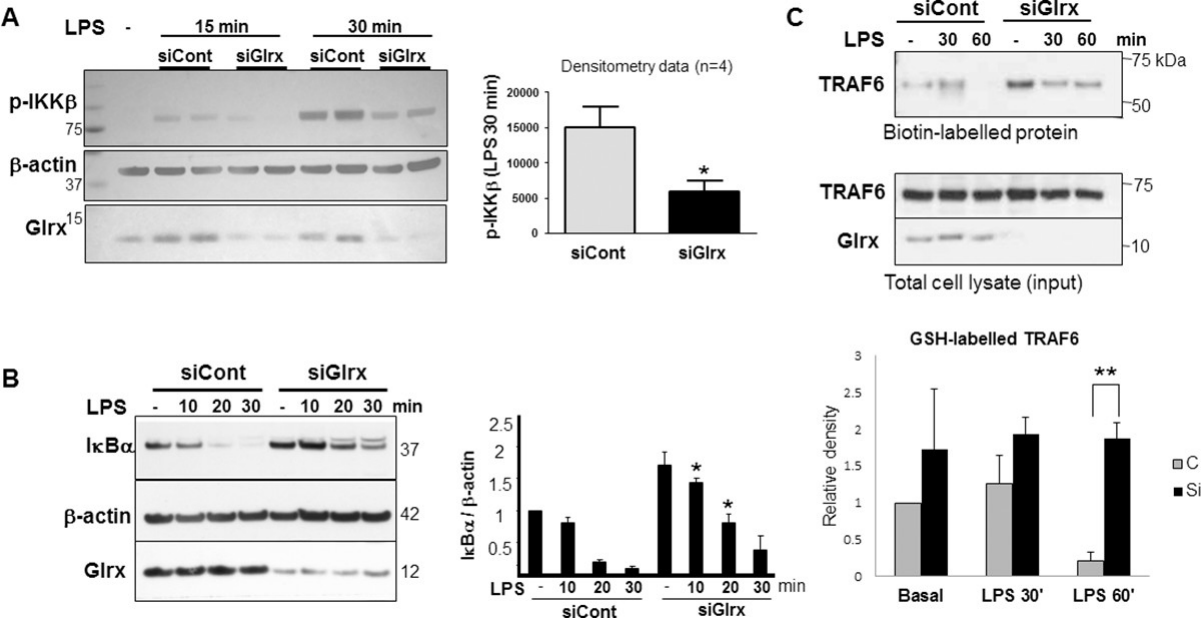
LPS-induced NF-κB is inactivated by Glrx knockdown via TRAF6–IKKβ inactivation. **(A)** LPS (100 μg/ml)-induced phosphorylation of IKKβ (15 min, 30 min) was attenuated in RAW267.4 cells treated with siGlrx. The representative Western blot and densitometry analysis from 4 independent experiments are shown. *p<0.05 compared to control. (**B)** Western blotting show that LPS (100 μg/ml)-induced IκBα degradation was inhibited by knockdown of Glrx. Densitometric analysis of 3 experiments shows significant retention of IκBα which may attenuate NF-κB nuclear translocation. (**C)** RAW267.4 cells were treated with siCont or siGlrx for 2 days prior to the experiment. Biotin-GSH ester was loaded for 2 hours in RAW267.4 cells with or without LPS for the indicated times. Biotin-labeled proteins were pulled down, separated by SDS-PAGE and blotted with TRAF6 antibody. Details are described in Methods. Representative data showing GSH-TRAF6 adducts are shown in the upper panel. TRAF6 and Glrx expression in whole cell lysate are shown in the lower panel. Densitometry of GSH-labelled TRAF6 was assessed as basal conditions (no LPS) of control cells set at 1. The graph shows the average of 3 separate experiments (n = 3, * P<0.05). Retention of GSH adducts on TRAF6 indicates impaired activation of TRAF6 [33].

Glrx inhibition may inactivate NF-κB via GSH adducts on IKKβ. We tested the NF-κB signaling pathways and found that LPS-induced phosphorylation of IKKβ was significantly attenuated following Glrx knockdown in RAW264.7 cells (**Fig 5A**). IKKβ phosphorylates IκBα and results in its degradation and nuclear translocation of NF-κB subunits. As expected, LPS-induced degradation of IκBα was significantly inhibited by Glrx knockdown (**Fig 5B**). We next examined TRAF6, further upstream of IKKβ.

### GSH adduct formation of TRAF6 was prolonged in RAW264.7 cells with Glrx knockdown

It has been shown that TNF receptor-associated factor 6 (TRAF6) is modified with GSH adducts and its ubiquitin ligase activation requires Glrx [33]. TRAF6 is an upstream signaling molecule of IKKβ. To detect GSH adducts formation on TRAF6, RAW264.7 cells were incubated with biotinylated GSH ester followed by pull down with Streptavidin beads. TRAF6 was detected as a GSH-labeled protein, and the GSH-TRAF6 adducts tended to be increased in Glrx knockdown cells under basal conditions. LPS treatment removed GSH adducts following 60 minutes in control cells, but, at this time, GSH adducts remained in Glrx knockdown cells (**Fig 5C**). This data indicates that endogenous Glrx regulates GSH adducts on TRAF6 and its activation in response to TLR4 activation.

### Exogenous IL-33 induces Glrx and IL-33 in macrophages

**Fig 6:**
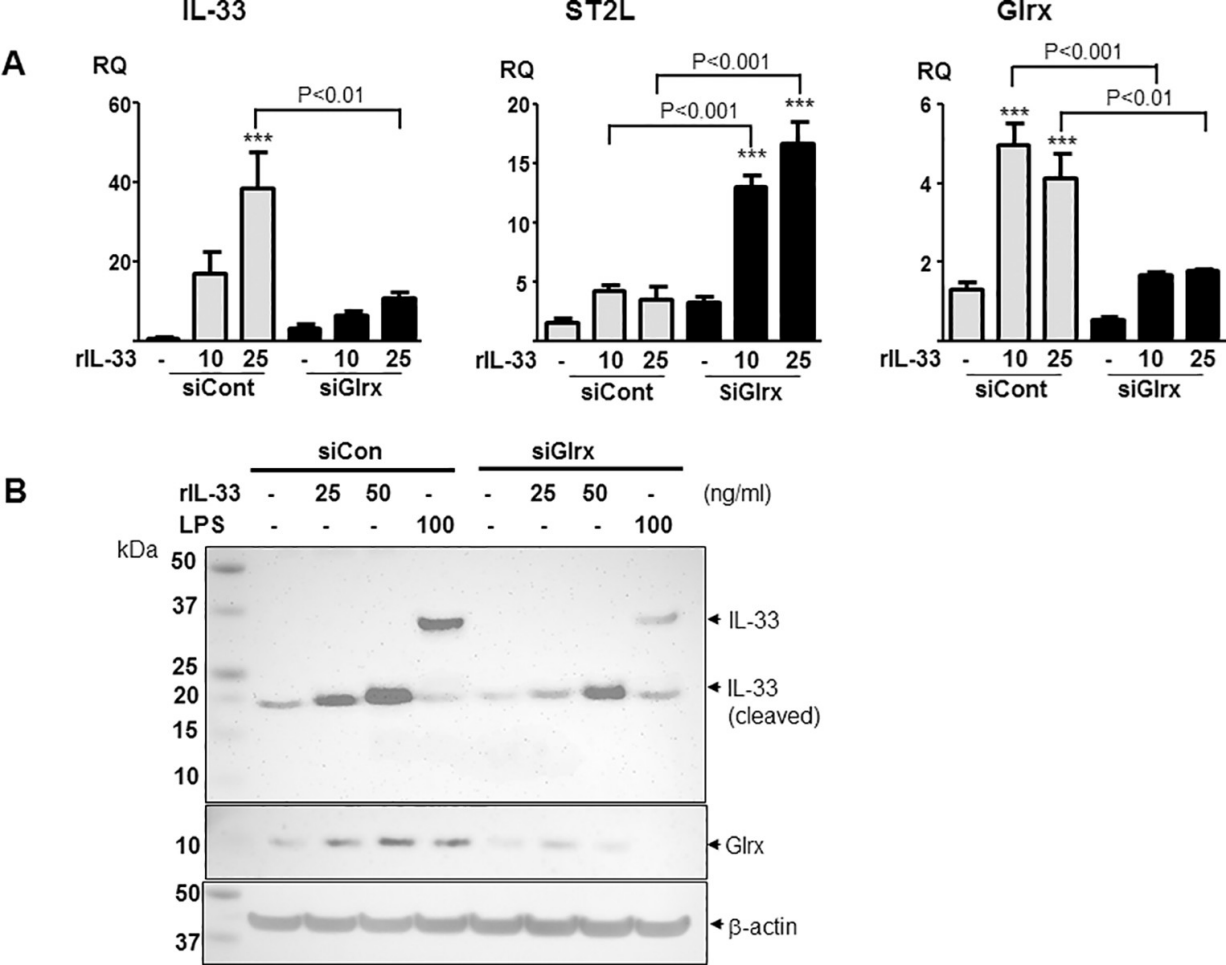
Exogenous IL-33 induces Glrx and IL-33 in macrophages. RAW267.4 cells were stimulated by rIL-33 (10, 25 ng/ml) for 6 hrs after treatment with siCont or siGlrx. (**A)** Gene expression of IL-33, Glrx, and ST2L was assessed by RT-qPCR. (**B)** rIL-33-induced IL-33 protein was truncated form compared to LPS-induced IL-33, and induction was attenuated by siGlrx. ***p<0.001 shows difference from control (no IL-33) in each siRNA group.

The relation between exogenous IL-33 and endogenous IL-33 induction is not known. Since TRAF6 is essential for IL-33 signaling [34] and expression of its receptor, ST2L, is upregulated by Glrx knockdown, the effects of IL-33 on the induced expression of IL-33, ST2L, and Glrx were examined in RAW264.7 cells. Recombinant IL-33 (rIL-33) (10–25 ng/ml) induced mRNA of Glrx as well as IL-33, and Glrx inhibition significantly attenuated this induction (**Fig 6A**). In contrast, ST2L was upregulated by Glrx knockdown in the presence of IL-33 similar to this observation following LPS stimulation (**Figs 1 and 2**). Interestingly, rIL-33-induced IL-33 protein was found to be of smaller size (20 kDa), which may be a cleaved, potent form [35,36], not the full-length form of IL-33 (~30 kDa) that is induced in response to LPS (**Fig 6B**). These data suggest that LPS and IL-33 share the common signaling pathway via Glrx-regulated TRAF6-NF-κB activation leading to IL-33 production in macrophages.

### Endogenous IL-33 *in vivo* is required for Glrx protein induction in cockroach antigen (CRA)–stimulated mouse lungs

**Fig 7:**
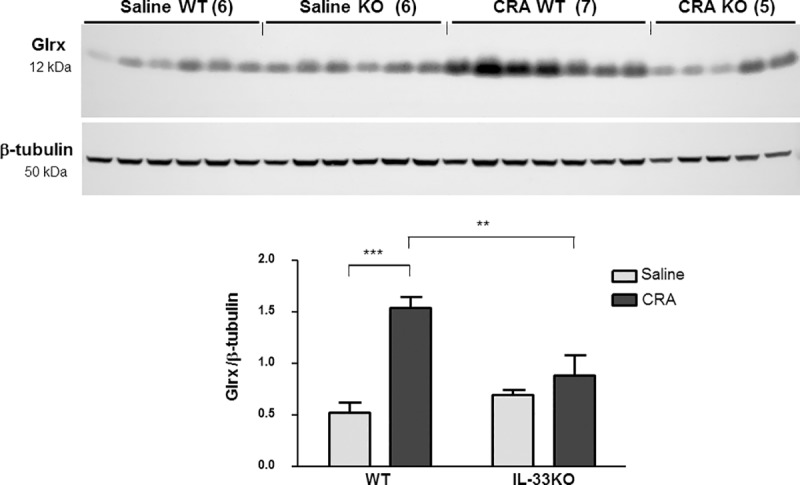
Glrx expression in cockroach antigen (CRA)-induced allergic lungs. WT and IL-33 KO mice were treated with CRA as described in the methods. Glrx protein expression in the lungs was examined by Western blotting. Relative expression of Glrx to β-tubulin by densitometry was compared between Saline and CRA treated lungs. Expression of Glrx protein in response to CRA was diminished in IL-33 KO mice compared to WT mice. (n = 5–7 mice/group, **p<0.01, ***p<0.001).

In CRA-stimulated mouse lungs from WT and IL-33 KO mice, protein levels of Glrx were increased compared to saline-treatment in WT mice in response to CRA, but Glrx induction was attenuated in lungs from IL-33 KO mice (**Fig 7**). This indicates that endogenous IL-33 *in vivo* promotes Glrx induction following allergen stimulation.

## Discussion

Protein-glutathione (GSH) adducts, or *S*-glutathionylation, are oxidative thiol modifications that may alter cellular protein function including signaling and transcriptional activity. Glutaredoxin-1 (Glrx) has been recognized as an important regulator of redox signaling by controlling levels of protein-GSH adducts in various pathophysiological conditions. The bacterial endotoxin, LPS, significantly induced Glrx expression in macrophages, which activated NF-κB likely through reversing GSH adducts (de-glutationylation) [26,27]. Our data further indicate that in macrophages LPS-induced Glrx plays an important role in TRAF6 activation, the upstream signaling molecule of IKKβ leading to NF-κB activation. This study demonstrates the novel role of cellular Glrx on the regulation of IL-33 production via TRAF6 in LPS-stimulated macrophages. Furthermore, we demonstrate that administration of rIL-33 in macrophages results in increased expression of both IL-33 and Glrx, and this is pathway is also dependent on Glrx.

TRAF6 is an adaptor molecule essential for MyD88-dependent TLR4 signaling [37] as well as IL-33 signaling [20,34]. TRAF6 is an E3 ubiquitin ligase that causes Lys-63-linked auto-poly-ubiquitination leading to activation of both IKK and mitogen-activated protein kinases (MAPKs) pathways. Chantzoura et al. show that TRAF6 is modified with the addition of GSH adducts at the amino terminal RING domain, the site of E3 ubiquitin ligase activity [33]. Glrx-dependent removal of GSH on TRAF6 is crucial for auto-polyubiquitination and subsequent activation of IKKβ in response to IL-1 in HEK293 or HeLa cells [33]. Consistent with their report, we demonstrated that TRAF6 was *S*-glutathionylated under basal conditions in macrophages and this was reversed by LPS stimulation. This is similar to β-actin, which undergoes deglutathionylation in response to growth factor stimulation leading to functional changes including actin polymerization and reorganization [38]. Our data also confirm that Glrx was responsible for deglutathionylation because GSH adducts on TRAF6 remained after LPS stimulation under Glrx knockdown conditions. Glrx itself is an NF-κB-responsive gene [26] and its expression increases following LPS stimulation, but the mechanism by which Glrx is activated within one hour remains unclear. Although LPS-induced IL-33 induction is almost completely blocked by the NF-κB inhibitor, JSH23, in our experiments, Glrx induction was only partially inhibited. Other transcription factors including AP-1 [39] may regulate LPS-induced Glrx transcription.

Furthermore, IL-33 downstream signaling requires TRAF6 [20,34,40]. Therefore, when TRAF6 is inactivated by Glrx knockdown, IL-33 signaling as well as its production are limited. We found that exogenous rIL-33 induced Glrx and also IL-33 mRNA was dependent on Glrx expression in macrophages. Interestingly, rIL-33 induced IL-33 protein as a short, cleaved form. Unlike IL-1β, full-length IL-33 is active without processing by caspases [41,42]. It has been recently shown, however, that elastase or proteases cleave IL-33 to smaller forms that possess much higher bioactivity compared with the full-length IL-33 [35,36]. In addition, the short form has cysteine residues that can form disulfides by oxidation and result in a shift on the gel [43]. We confirmed the shift in a non-reduced gel (**S2 Fig**) but Glrx knockdown did not change the shift or size of IL-33. Therefore, Glrx likely controls IL-33 induction at transcriptional levels. The reason why rIL-33 induced a cleaved form of IL-33 is unknown and requires further investigation.

The expression of the IL-33 receptor, ST2L (IL1RL1), was elevated in Glrx knockdown macrophages. LPS did not show additional increase in Glrx KO mouse macrophages (Fig 1), but its expression was enhanced further in RAW cells with siGlrx (Fig 2). The difference in LPS response may arise from either cell type and/or complete Glrx deletion versus partial Glrx inhibition. In RAW cells, either LPS or IL-33 upregulated ST2L expression under Glrx knockdown conditions (Fig 2 and Fig 6). We speculate that TRAF6 signaling negatively regulates ST2L expression, and TRAF6 inhibition by Glrx knockdown may activate ST2L induction. It is reported that Th2 stimulation enhanced ST2L expression while IFN-gamma decreased it in T cells [44]. The transcriptional regulation of ST2L has to be further explored. Notably, IL-33 signaling is impaired in Glrx knockdown cells, therefore upregulated ST2L could be a compensatory response to the diminished IL-33 axis.

Our findings are highly relevant to conditions that result in oxidative stress such as cigarette smoke that decreases Glrx mRNA and protein expression [45], which may lead to lower production of IL-33, and in addition may oxidize IL-33 to be inactivated [43]. IL-33 is beneficial in the setting of wound healing [16] but exacerbates allergic inflammation in some models of asthma [46]. Therefore, regulation of IL-33 by Glrx can be beneficial or deleterious depend on the pathological conditions.

As we found the link between Glrx and IL-33 signaling in macrophages, we extended the findings to examine expression of Glrx and IL-33 in a relevant mouse model of asthma. Cockroach antigen (CRA)-stimulated lungs expressed high levels Glrx protein compared to saline treated lungs. Glrx expression was attenuated in IL-33 KO mouse lungs. Although lungs contain various types of cells other than macrophages, this data suggests IL-33 may promote Glrx expression and inflammatory reaction in allergic lungs. Also, we tested CRA-induced Glrx mRNA in macrophages from IL-33 KO mice. We could detect CRA-induced Glrx at 6 hours, but it was not significantly inhibited in IL-33 KO cells (**S3 Fig**). This suggests that CRA-induced Glrx mRNA induction may not require IL-33 following short exposure. However, chronic stimulation *in vivo* may attenuate Glrx protein levels in the lung containing various cell types in the absence of IL-33. In addition, others have shown that Glrx expression is increased in ovalbumin-induced or house dust mite-induced allergic airway model in mice [47,48]. Increased Glrx further activates NF-κB and may promote IL-33 production as a recent report shows oxidative stress enhances IL-33 in human airway epithelial cells [49].

Furthermore, we tested CRA-induced IL-33 in RAW cells, and found CRA induced IL-33 protein (full length) at 6 hours but the expression was not inhibited with siGlrx (**S4 Fig**). We speculate that CRA signaling is different from LPS pathway and induces IL-33 in macrophages under inhibition of Glrx. In a similar allergic *in vivo* model, Glrx ablation attenuated Th2 cytokines (IL-13, IL-5) in the house dust mite-stimulated lungs although IL-33 levels were not reported [48]. CRA-induced IL-33 may be modulated in other types of cells (epithelial, endothelial) in the lungs of Glrx KO mice. Further investigation is required to clarify these issues.

In conclusion, LPS-induced Glrx activates NF-κB pathways by regulating GSH adducts on TRAF6, thereby contributing to IL-33 production in macrophages. Exogenous IL-33 also stimulates Glrx induction and active IL-33 production.

## Supporting information

S1 Fig F4/80 staining on mouse peritoneal macrophages. Adhered peritoneal cells were fixed and stained with anti-mouse F4/80 antibody (red) and Hoechst (blue). (PDF)

S2 Fig IL-33-induced IL-33 protein is shifted in non-reduced condition. LPS (100ng/ml) or IL-33 (50 ng/ml) was added in RAW cells for 6 hours, and cellular proteins were analyzed in reduced and non-reduced gel.
(PDF)

S3 Fig CRA-induced Glrx mRNA in WT and IL-33 KO mouse macrophages. CRA (Cockroach antigen 100 μg/ml) or PBS was added in isolated mouse macrophages from WT and IL-33 KO mice. After 6 hours RNA was isolated from cells by Trizol and Glrx expression was examined (n = 3–4 wells).
(PDF)

S4 Fig CRA-induced IL-33 in RAW cells. Different doses of CRA (0–80 μg/ml) was tested to examine IL-33 and Glrx induction in RAW cells with siControl or siGlrx RNA.
(PDF)
