# Supplementary figures and images for: IL-33 induction and signaling are controlled by glutaredoxin-1 in mouse macrophages
Source: PLoS One. 2019 Jan 25;14(1):e0210827. doi: 10.1371/journal.pone.0210827 (PMC6347181; doi:10.1371/journal.pone.0210827)

## S1. F4/80 staining on mouse peritoneal macrophages

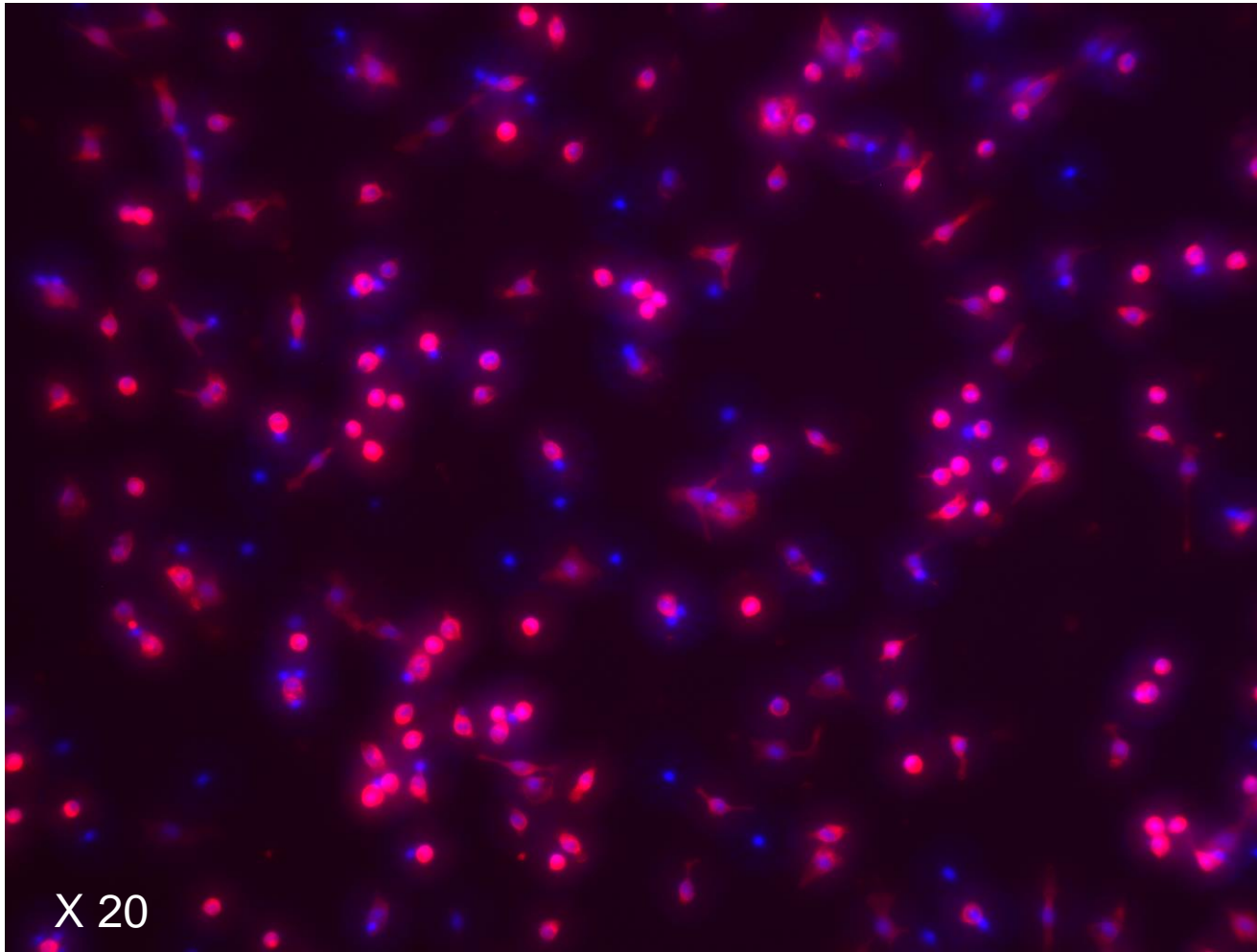

Red: anti-F4/80 antibody

Blue: Hoechst

Supplement: S1 Fig — (PDF) [file pone.0210827.s001.pdf]

## S2. IL-33-induced IL-33 protein is shifted in non-reduced condition

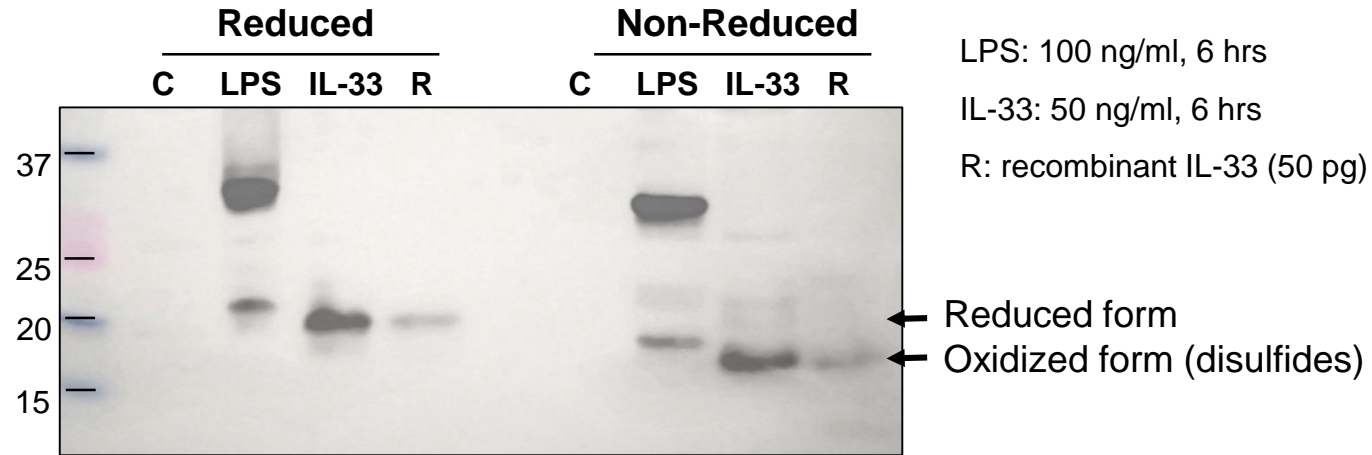

Supplement: S2 Fig — LPS (100ng/ml) or IL-33 (50 ng/ml) was added in RAW cells for 6 hours, and cellular proteins were analyzed in reduced and non-reduced gel. (PDF) [file pone.0210827.s002.pdf]

### S3. CRA-induced Glrx mRNA in WT and IL-33 KO mouse macrophages

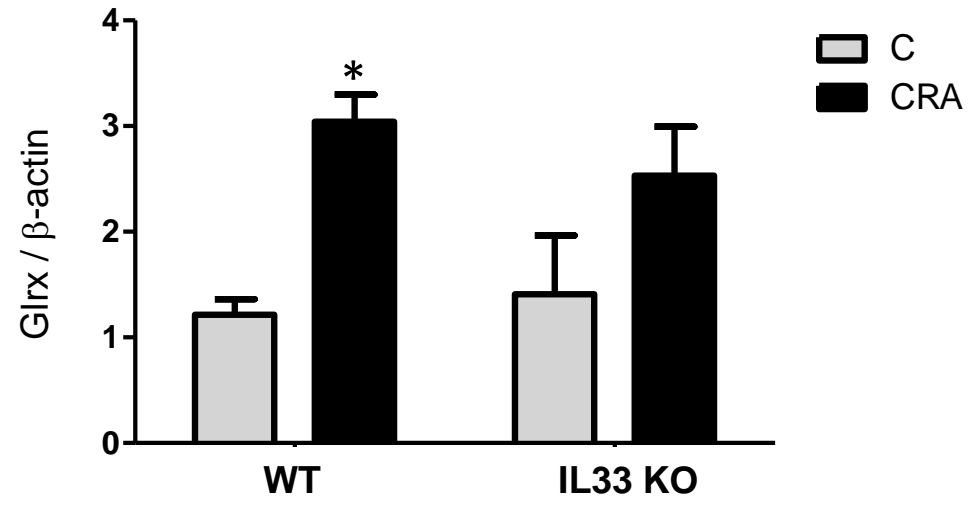

\* P<0.05 from WT control

Supplement: S3 Fig — CRA (Cockroach antigen 100 μg/ml) or PBS was added in isolated mouse macrophages from WT and IL-33 KO mice. After 6 hours RNA was isolated from cells by Trizol and Glrx expression was examined (n = 3–4 wells). (PDF) [file pone.0210827.s003.pdf]

#### S4. CRA-induced IL-33 in RAW cells

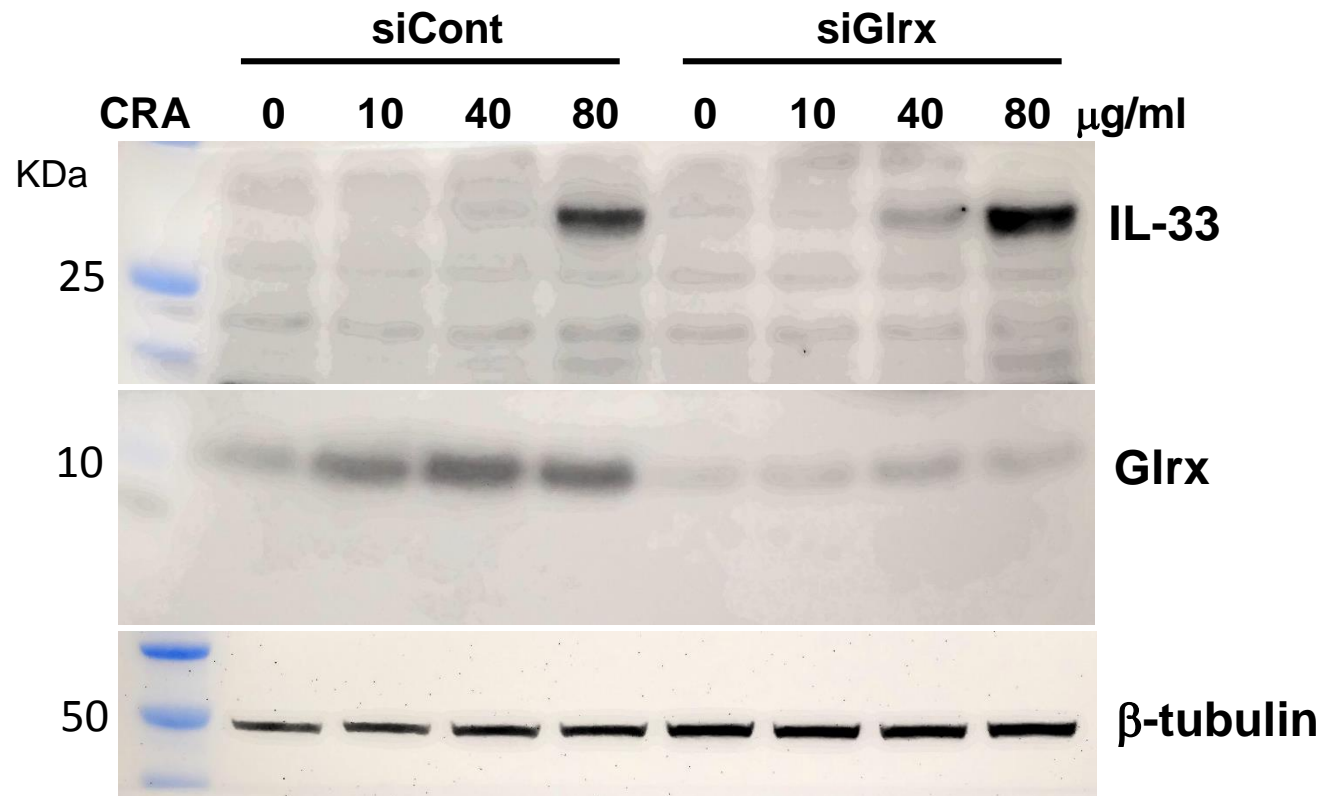

Supplement: S4 Fig — Different doses of CRA (0–80 μg/ml) was tested to examine IL-33 and Glrx induction in RAW cells with siControl or siGlrx RNA. (PDF) [file pone.0210827.s004.pdf]
